# Evaluation of the Factors Associated with Reinfections towards SARS-CoV-2 Using a Case Control Design

**DOI:** 10.3390/jcm12113861

**Published:** 2023-06-05

**Authors:** Giuseppe La Torre, Gianluca Paglione, Lavinia Camilla Barone, Vittoria Cammalleri, Augusto Faticoni, Mattia Marte, Roberta Noemi Pocino, Carlo Maria Previte, Andrea Bongiovanni, Corrado Colaprico, Eleonora Ricci, Valentin Imeshtari, Maria Vittoria Manai, David Shaholli, Vanessa India Barletta, Giovanna Carluccio, Luca Moretti, Francesca Vezza, Lorenzo Volpicelli, Anna Paola Massetti, Lilia Cinti, Piergiorgio Roberto, Anna Napoli, Guido Antonelli, Claudio Maria Mastroianni, Sabina Sernia

**Affiliations:** 1 Department of Public Health and Infectious Diseases, Sapienza University of Rome, 00185 Rome, Italy; 2 Department of Molecular Medicine, Sapienza University of Rome, 00185 Rome, Italy

**Keywords:** reinfections, SARS-CoV-2, case-control, contact tracing

## Abstract

Objective: The risk of reinfection with SARS-CoV-2 has been rapidly increased with the circulation of concerns about variants. So, the aim of our study was to evaluate the factors that increase the risk of this reinfection in healthcare workers compared to those who have never been positive and those who have had only one positivity. Methods: A case-control study was carried out at the Teaching Hospital Policlinico Umberto I in Rome, Sapienza University of Rome, in the period between 6 March 2020 and 3 June 2022. Cases are healthcare workers who have developed a reinfection with the SARS-CoV-2 virus, while controls were either healthcare workers who tested positive once or those who have never tested positive for SARS-CoV-2. Results: 134 cases and 267 controls were recruited. Female gender is associated with a higher odds of developing reinfection (OR: 2.42; 95% CI: 1.38–4.25). Moreover, moderate or high alcohol consumption is associated with higher odds of reinfection (OR: 1.49; 95% CI: 1.19–1.87). Diabetes is also associated with higher odds of reinfection (OR: 3.45; 95% CI: 1.41–8.46). Finally, subjects with increased red blood cell counts have higher odds of reinfection (OR: 1.69; 95% CI: 1.21–2.25). Conclusion: From the prevention point of view, these findings indicate that particular attention should be paid to subjects with diabetes mellitus, women and alcoholic drinkers. These results could also suggest that contact tracing represents a fundamental approach model against the SARS-CoV-2 pandemic, together with the health information of participants.

## 1. Introduction

SARS-CoV-2 is the responsible biological agent of COVID-19. It was declared a public health emergency in January 2020 and then a pandemic in March of 2020. Against infection with the SARS-CoV-2 virus, it is the adaptive immune response that should deliver long-term protection. The efficiency and persistence of natural protective immunity caused by the coronavirus (SARS-CoV-2) infection or vaccination are currently unknown. Reinfection cases have been reported in different countries, although the differentiation between cases of reinfection and viral persistence remains a challenge [1].

SARS-CoV-2 is predisposed to genetic evolution while adapting to its hosts with the development of mutations over time, resulting in the emergence of multiple variants. With the emergence of multiple variants, the CDC and the WHO established a classification system for distinguishing the emerging variants of SARS-CoV-2 into variants of concern (VOCs) and variants of interest (VOIs).

VOCs are associated with enhanced transmissibility or virulence, reduction in neutralization by antibodies obtained through natural infection or vaccination, the ability to evade detection, or a decrease in therapeutics or vaccination effectiveness. Based on the recent epidemiological update by the WHO, as of 11 December 2021, five SARS-CoV-2 VOCs have been identified since the beginning of the pandemic:

1. Alpha (B.1.1.7): first variant of concern described in the United Kingdom (UK) in late December 2020
2. Beta (B.1.351): first reported in South Africa in December 2020
3. Gamma (P.1): first reported in Brazil in early January 2021
4. Delta (B.1.617.2): first reported in India in December 2020
5. Omicron (B.1.1.529): first reported in South Africa in November 2021 [2]

Based on current knowledge, the risk of reinfection for healthcare workers is heterogeneous, with a 10-fold difference (from 0.21 to 2.3%) [3,4]. The characteristics of healthcare workers reinfected with SARS-CoV-2 indicate that they are mainly females (71–80%), nurses, immunosuppressed, 39.2 years old, with kidney disease and previously infected with mild SARS-CoV-2 [4,5,6].

For the reinfection, the CDC’s criteria are:

- A subsequent RT-PCR positive to SARS-CoV-2 > 45 days after the initial presentation if the second test is accompanied by compatible symptoms or epidemiological exposure;
- A subsequent RT-PCR positive to SARS-CoV-2 > 90 days after the initial presentation if the second test is performed among an asymptomatic HSM with close contact with a person known to have a laboratory-confirmed COVID-19. Thereafter, ‘probable reinfection’, defined by clinical context (symptoms, risk exposure) plus a Cycle Threshold (Ct) < 37 and the absence of other diagnoses, was assessed by an infectious disease specialist and a microbiologist [6].

A systematic review carried out by Gopinath [3] shows that SARS-CoV-2 reinfections are seen due to prolonged exposure, predominantly in healthcare workers despite vaccination. From the clinical point of view, most reinfected patients show clinical symptoms, but they were predominantly mild [4]. There is evidence that only a small number of studies reported patients being asymptomatic at both the first and secondary infection [7].

In Italy, from 24 August 2021 to 5 June 2022, 532,755 cases of reinfection were reported, equal to 4% of the total number of notified cases. In the last week, the percentage of reinfections, out of the total number of reported cases, was 7.4%, an increase compared to the previous week, in which the figure was 6.3% [8].

The analysis of the risk of reinfection, starting from 6 December 2021, the date considered to be the reference date for the start of the spread of the Omicron variant, shows an increase in the adjusted relative risk of reinfection (values significantly greater than 1):

- In subjects with the first diagnosis of COVID-19 notified for more than 210 days compared to those who had the first diagnosis of COVID-19 between the previous 90 and 210 days;
- In subjects not vaccinated or vaccinated with at least one dose for over 120 days compared to vaccinated with at least one dose within 120 days;
- In females compared to males. The greater risk in female subjects may probably be due to the greater presence of women in the school setting (>80%), where intense screening activity is carried out, and to the fact that women perform the role of caregiver in the field more frequently;
- In the younger age groups (from 12 to 49 years) compared to people with the first diagnosis between the ages of 50–59 years. Probably the greater risk of reinfection in the younger age groups is attributable to behaviors and exposures at greater risk, compared to the age groups over 60 years;
- In healthcare workers compared to the rest of the population [8].

There is already evidence that patients with chronic diseases, such as chronic renal failure, cardiovascular disease, bronchopneumopathy, neuropathy and autoimmune diseases are at increased risk of reinfection [3,7,9], and that a significant rise in SARS-CoV-2 reinfection rate has occurred in vaccinated healthcare workers during the omicron wave [10].

The aim of our study was to evaluate the factors that increase the risk of reinfections for SARS-CoV-2 in the healthcare workers compared to those who have had only one SARS-CoV-2 positivity and who have never been positive.

## 2. Materials and Methods

### 2.1. Study Design

The work under review consists of a single centre case-control study involving two different control groups.

### 2.2. Participants

We considered the subjects who tested positive for the SARS-CoV-2 virus, among the health workers of the Teaching Hospital Policlinico Umberto I in Rome, Sapienza University of Rome, in the period between 6 March 2020 and 3 June 2022.

Participants in the study were represented by cases, i.e., healthcare workers who have developed a reinfection with the SARS-CoV-2 virus, and by two control groups, the first of which was represented by healthcare workers who tested positive, once only, while the second control group was made up of healthcare workers who have never tested positive for SARS-CoV-2. The choice of two different control group was made to increase the power of the study.

The criteria for pairing, between cases and controls, included age, gender and profession.

Each case of double positivity to the SARS-CoV-2 virus was matched to two controls, one of which belongs to the control group which includes the subjects who tested positive once and the other belonging to the control group which includes subjects who have never had a positive response to the SARS-CoV-2 virus. In the teaching hospital, since the start of the pandemic, a bi-weekly surveillance via PCR testing was carried out among the HCWs. Moreover, serology was carried out twice, one in the period from May to June 2020, and the other during a routine health check at the Occupational Medicine Unit.

### 2.3. Variables

The variables considered for carrying out the study are: age (continuous variable), gender (dichotomous variable), profession (nominal variable), habits (dichotomous variables) such as smoking and alcohol consumption, physical activity, diseases such as diabetes, hypertension and obesity (assessed by the latest available BMI), the haematochemical parameters (red blood cells, hemoglobin, platelets, absolute white blood cell count), drugs taken, existing occupational hazards (biological hazard due to the exposure with human bodily matter, such as blood, tissue, saliva, mucus, urine and faeces; manual handling of loads, intended as lifting, holding, putting down, pushing, pulling, carrying or moving of a load; night shift work; exposure to low-dose ionizing radiations for interventional physicians, radiologists, radiation oncologists and associated technical staff). All the variables were assessed at the last health surveillance visit. 

The ADVIA^®^ 2120i Hematology System was used for the evaluation of the haematochemical parameters. Alcohol consumption was measured using the Audit-C (Alcohol Use Disorders Identification Test for screening for unhealthy alcohol use). Physical activity was classified as follows: No physical activity, <4 h per week, 4 < h < 8 per week, > 8 h per week. All other data (clinical data and occupational exposure) were derived from the clinical chart of the HCW. 

In our study to diagnose the infection, we considered positivity on the RT-PCR test for SARS-CoV-2 with Ct < 35, rapid antigenic buffer or third generation antigenic buffer with COI (Cut Off Index) > 1

All those who tested negative to the third generation molecular or antigenic buffer with a COI index < 1 were considered healed.

The purpose of our study was to identify any factors associated with SARS-CoV-2 reinfection in health workers at the Teaching Hospital Policlinico Umberto I in Rome.

Data of health workers who tested positive for the SARS-CoV-2 virus were obtained from the health surveillance database of health workers who tested positive for the SARS-CoV-2 virus of the Policlinico Umberto I in Rome, while the data relating to the subjects never tested positive were collected from the general database of the Occupational Medicine Service of the Policlinico Umberto I in Rome. All data concerning lifestyle, hematological parameters, chronic conditions and workplace risks were derived from the personal chart of each HCW. 

In this study, we considered all cases of SARS-CoV-2 reinfection that we are aware of, using a 1:2 ratio for case controls. In matching cases (reinfections) with controls (never infected and single infection), we considered the same period of occurrence of the infections and the visit for health check surveillance, for taking under control the waves of SARS-CoV-2 variants circulating. It is interesting to note that only three reinfections occurred in the period between 10 October 2020 and 23 July 2021, while all others occurred after 9 December 2021.

### 2.4. Statistical Analysis

Sample size calculations were based on the following assumptions:

- Confidence level: 95%
- Power: 80%
- Expected frequency of chronic disease among controls: 20%
- Odds ratio to detect: 2.

The calculations were made using the package Statcalc of EpiInfo. According to the above parameters, we should have recruited 122 cases and 244 controls. In order to take into account possible refusals to participate, an increase of 10% was added, so the final sample to be recruited comprised 134 cases and 168 controls. 

Statistical analysis involves the use of descriptive and inferential statistics. Descriptive sample statistics were performed using the mean and standard deviation (SD) for quantitative variables, while frequencies and percentages were calculated for qualitative variables.

A univariate analysis was performed using the chi-square test, to evaluate the differences between groups (cases and controls) for the categorical variables, instead the T-Student test or the Mann–Whitney test to compare the groups for the quantitative variables, with normal or non-normal distribution, respectively.

A multivariate analysis was conducted using the multiple logistic regression model, with multiple variables simultaneously and of a quantitative type. Two types of models were carried out: a full model with all variables and a stepwise approach (backward elimination procedure). A stratified analysis was also carried out to verify if gender can act as an effect modifier.

The regression results will be represented as odds ratios (OR) and 95% confidence intervals (95% CI).

In these models, the single dichotomous variable was used as the dependent variable, while all the others were considered covariates.

All statistical analyses were performed using SPSS (Statistical Package for Social Science) for Windows 25.0 (IBM, Armonk, NY, USA).

Statistical significance was set at a *p*-value of less than 0.05.

## 3. Results

The characteristics of the participants are summarized in Table 1. The sample under examination is represented by a population of 401 health care workers containing up of 250 females (62.3%) and 151 males (37.7%), with an average age of 43.03 years.

The professions are represented as follows:

- 142 physicians (35.3%);
- 182 nurses (45.3%);
- 10 technicians (2.5%);
- 17 administrative (4.2%);
- 51 healthcare assistants (12.7%).

Non-smokers are 62.4% of the sample, compared to smokers, who make up 37.6%.

Concerning the consumption of alcohol, the subjects who do not consume alcohol are 31.3%, while 13.6% have an Audit-C score greater than or equal to 3 points, corresponding to an average–high consumption of alcohol.

The subjects in the sample have a high percentage of sedentary lifestyle; in fact, 44.9% say they do not engage in physical activity.

Diabetic patients represent 6.6% of the sample, in line with the Italian national average of the prevalence of the disease.

Hypertensive subjects make up 14% of the sample. 45.4% take drugs for any reason.

With regard to the tasks performed, 96.4% of the sample is exposed to biological hazard, 49% perform manual handling loads and 77.4% perform night shifts while 14.3% are exposed to ionizing radiations.

The mean value of BMI (Body Mass Index) is 24.53. For red blood cells, it is 4.40 (×10^12^/L). For haemoglobin, it is 14·18 g/dL. For platelets, it is 242.72 (×10^9^/L). For white blood cells, it is 6.04 (×10^9^/L).

### 3.1. Univariate Analysis

#### 3.1.1. Reinfection vs. All Controls

As can be seen from Table 2, with regard to alcohol intake, the subjects have a consumption equal to or greater than 3 points and 21.5% of reinfection cases compared to 9.7% of all controls, with a statistical significance *p* = 0.002. Therefore, moderate or high intake (greater than or equal to 3 points) is significantly associated with the development of double positivity in relation to all controls.

With regard to diabetes mellitus, 13% of reinfection cases and 3.4% of all controls are affected, with a statistical significance *p* < 0.001.

The red blood cells, in reinfections, appear to be increased (4.80 × 10^12^/L), compared to controls (4.17 × 10^12^/L), with a statistical significance *p* <0.001.

Finally, the white blood cells in reinfections are increased (6.52 × 10^9^/L) compared to controls (5.78 × 10^9^/L), with a statistical significance *p* = 0.007.

No difference was found concerning the vaccination against COVID-19.

#### 3.1.2. Reinfection vs. Never Positive 

In addition, regarding the comparison between cases of reinfection against the control group of subjects who have never had evidence of positivity (Table 3), there is statistical significance in alcohol consumption with a score equal to or greater than 3 points for 21.5% of reinfection cases compared to 5.1% of never positive controls (*p* < 0.001).

Regarding diabetes, patients with reinfection are 13% while among the never positive controls they are 0.7%, statistically significant (*p* < 0.001).

Lastly, also in this case, the red blood cells, in reinfections, are increased (4.80 × 10^12^/L), compared to never positive controls (3.52 × 10^12^/L), with statistical significance *p* < 0.001, as well as white blood cells, which, in reinfections, are increased (6.52 × 10^9^/L) compared to never positive (5.06 × 10^9^/L), with statistical significance *p* < 0.001.

#### 3.1.3. Reinfection vs. Single Positivity

From the comparison between cases of reinfection and controls with single positivity, it is clear (Table 4) that there is a difference, not statistically significant, between the consumption of alcohol and diabetes mellitus.

Regarding the value of red blood cells and white blood cells, there is no difference between cases of reinfection and controls with single positivity.

### 3.2. Multivariate Analysis

#### 3.2.1. Reinfection vs. All Controls

In multivariate analysis, relative to the comparison, between subjects with reinfection and all controls (Table 5), it appears that the female gender is associated with a higher probability of developing reinfection (O.R: 2.42; 95% CI: 1.38–4.25) than the male gender.

Regarding alcohol consumption, there is a higher risk of reinfection (OR: 1.49; 95% CI: 1.19–1.87) among subjects with moderate or high intake (greater than or equal to 3 points), compared to those who do less use.

Diabetes is associated with a higher risk of reinfection (OR: 3.45; 95% CI: 1.41–8.46) than in non-diabetic patients.

Subjects with increased red blood cell counts have a higher risk of reinfection (OR: 1.69; 95% CI: 1.21–2.25).

Stratifying the analysis by gender, we found that this factor acts as an effect modifier. In fact, the reinfection is associated among females to alcohol consumption (OR = 1.58; 95%CI: 1.16–2.15) and to number of red blood cells (OR = 1.77; 95%CI: 1.16–2.69). On the other hand, among males this association was found for diabetes (OR = 8.57; 95%CI: 1.59–46.3), alcohol consumption (OR = 1.56; 95%CI: 1.10–2.21), number of red blood cells (OR = 2.52; 95%CI: 1.33–4.79), number of white blood cells (OR = 0.70; 95%CI: 0.53–0.93) and hemoglobin level (OR = 0.54; 95%CI: 0.32–0.90). 

#### 3.2.2. Reinfection vs. Never Positive

Concerning the comparison between subjects with reinfection and never positive ones (Table 6), the gender variable shows an increased risk of reinfection for the female gender (OR: 3.42; 95% CI: 1.66–7.03), compared to males.

Alcohol consumption also confirms an increased risk of reinfection in this comparison with OR: 1.51; 95% CI: 1.13–2.01.

Diabetic patients have a greatly increased risk of reinfection (OR: 15.42; 95% CI: 1.92–123.60).

The increased number of red blood cells is also associated with an increased risk of reinfection (OR: 2.47; 95% CI: 1.63–3.74).

Stratifying the analysis by gender, we found that this factor acts as an effect modifier. In fact, the reinfection is associated among females only to number of red blood cells (OR = 2.30; 95%CI: 1.39–3.79). On the other hand, among males this association was found for diabetes (OR = 16.38; 95%CI: 1.54–174.2), alcohol consumption (OR = 1.92; 95%CI: 1.20–3.06), number of red blood cells (OR = 3.22; 95%CI: 1.64–6.32) and number of white blood cells (OR = 0.63; 95%CI: 0.43–0.91).

#### 3.2.3. Reinfection vs. Single Positivity 

By comparing the cases of reinfection with subjects who have developed a single positivity (Table 7), the association of the female gender with a greater risk of a second positivity is eliminated.

Additionally, in this case there is a greater risk of reinfection linked to medium or high alcohol consumption, greater than or equal to 3 points (OR: 1.40; 95% CI: 1.09–1.79).

Diabetic disease remains a risk factor for reinfection also in this comparison (OR: 4.39; 95% CI: 1.41–13.67).

Stratifying the analysis by gender, we found that this factor acts as an effect modifier. In fact, the reinfection is associated among females only to alcohol consumption (OR = 1.81; 95%CI: 1.26–2.62). On the other hand, among males this association was found for diabetes (OR = 8.24; 95%CI: 1.07–63.20) and alcohol consumption (OR = 1.46; 95%CI: 1.0–2.17).

**Figure 1:**
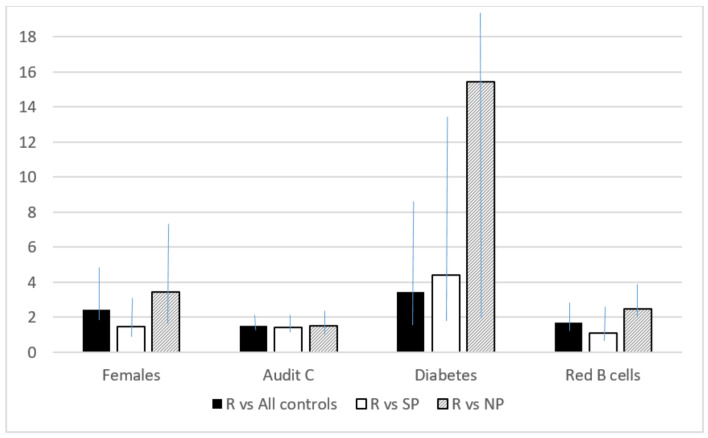
Summary plot of the results of multivariate analyses for different risk factors (results are expressed as Odds Ratios). Legend: R = reinfection; SP = single positivity; NP = never positivity.

In Figure 1 a summary plot of all the multivariate analysis can be seen. 

## 4. Discussion

In the present case control study, reinfection with SARS-CoV-2 appears to be associated with female gender, alcohol consumption, diabetes mellitus and an increase in the number of red blood cells.

In particular, the univariate analysis showed that from the comparison between all reinfection cases and all controls, statistical significance emerges for alcohol consumption, diabetes mellitus and the increase in the number of red and white blood cells in individuals who have been reinfected with the SARS-CoV-2 virus.

Still, in the univariate analysis, the comparison between reinfection cases and healthy controls, who have never become infected with the SARS-CoV-2 virus, showed an association between alcohol consumption, diabetes mellitus, an increased number of red and white blood cells and SARS-CoV-2 reinfection.

Additionally, the comparison, within the univariate analysis, between the cases of reinfection and the controls with single positivity, to the SARS-CoV-2 virus, has highlighted a difference, even not statistically significant, for the consumption of alcohol and diabetes mellitus, showing that both variables had a higher frequency in subjects with reinfection.

The same comparisons were performed in the multivariate analysis, which confirmed what emerged from the previous analysis, highlighting an increased risk of reinfection, with statistical significance, for the female gender.

This evidence was found in the comparison between the cases of reinfection and all the controls, as well as in that between the double positives and the never infected healthy controls.

Compared with other studies that have dealt with the relationship between COVID-19 and alcohol consumption, we have been able to observe how alcohol is related to an increased risk of infection with SARS-CoV-2. This has also been associated with lifestyle changes resulting from pandemic containment measures, with changes in alcohol consumption habits and with the increase in heavy drinking during the lockdown [11]. These associations can, in some way, be explained by evaluating the effects of alcohol on the immune system.

According to some studies, alcohol consumption, at any level, alters the host’s immune responses, modifying the number, phenotype and function of both innate and adaptive immunity cells. This impairment is exacerbated by the presence of comorbidities such as diabetes mellitus which in our study was found to be associated with an increased risk of reinfection [12].

However, further investigation is needed to understand in greater detail and completeness the relationship between alcohol consumption and reinfection with the SARS-CoV-2 virus.

Comparing the data obtained, in our analysis, on diabetes mellitus, with other studies [13], it was confirmed that there are more cases of reinfection with SARS-CoV-2 among diabetic patients because, in them, the immune system is dysfunctional, both as regards innate immunity and the adaptive response [14,15].

A systematic review by Azam et al. [16], in disagreement with what emerged in our work, highlights that individuals with diabetes are less likely to develop reinfection with SARS-CoV-2 (pooled RR = 0.52; 95%CI: 0.30–0.90). In the same review, it was also highlighted, in partial agreement with what we demonstrated, that subjects with low lymphocyte counts have a lower risk of reinfection (pooled RR = 0.58; 95%CI: 0.39–0.86), while our analysis showed that an increase in white blood cells is associated with an increased risk of reinfection.

In this regard, it would be interesting to understand whether the cases of reinfection with SARS-CoV-2 in subjects with diabetes are a consequence of the altered antibody production both after natural infection and after vaccination.

Therefore, in subjects with diabetes mellitus, there is a greater risk of reinfection with the SARS-CoV-2 virus, since the function of their immune system is impaired.

The analysis carried out by us confirms what has been reported by other studies about a higher rate of reinfection with SARS-CoV-2 in diabetic subjects and those of the female gender. Our results are in agreement with a recent systematic review, carried out by Ngugyen et al. [17], which demonstrates that being female and being a patient with comorbidities is associated with a higher risk of reinfection.

In the Mexican study by Murillo-Zamora [18], an association between some factors such as increasing age, obesity, diabetes mellitus and chronic kidney disease with a more severe form of symptomatic SARS-CoV-2 reinfection is described. In our work, however, a link between obesity and the risk of reinfection as well as with the variable age was not highlighted, even if it must be taken into account that our population was selected, and was relatively young, with an average age of 43 years, and therefore different from the general population considered in the cited work.

Relative to the observations regarding the female gender, a study in partial disagreement with what we have demonstrated highlighted a greater reinfection among males while for the female gender a higher rate of relapse was found, or reinfection with the same species or strain as SARS-CoV-2 [19]. Overall, however, and therefore in accordance with our evidence, without distinguishing between reinfection and relapse, the female gender shows higher odds of developing the SARS-CoV-2 reinfection. In this regard, it would have been interesting to know the difference between reinfection and relapse in relation to our cases; unfortunately, we do not have the data regarding the viral genotype.

In line with the data that emerged in our work, other studies also show that the female gender is more frequently involved in reinfections with SARS-CoV-2, although it is thought that women have a greater awareness of the importance of containment and distancing measures adopted to combat the current pandemic [20]. A possible explanation could include the hormonal differences existing between the two genders [21].

The SIREN study (SARS-CoV-2 Immunity and Reinfection Evaluation) [22], carried out with a large, multicentre, prospective cohort study involving 8278 health workers, belonging to the National Health Service of the United Kingdom, previously infected with SARS-CoV-2, found 153 cases of reinfection, a reinfection rate of 1.84%, a figure lower than that observed by us of 3.38%. The differences in reinfection rates can be attributed to the onset of new variants of the virus in the reporting period, such as Omicron, which also compromised the efficacy of currently available vaccines. From the same study, in accordance with our work, it emerged that the female gender is more associated with reinfection with SARS-CoV-2.

The finding of the increase in the number of red blood cells and white blood cells in cases of reinfection with SARS-CoV-2 can be explained by the reactive activation of erythroblasts and granuloblasts. This was also suggested by the hematologists of the Teaching Hospital Policlinico Umberto I, consulted by us, even if we have no scientific evidence in this regard. Another possible explanation for the relation between erythrocytosis and reinfection risk might be indirectly related to a lung disease or respiratory insufficiency. According to Huerga Encabo et al., the SARS-CoV-2 infection is directly or indirectly associated to an increase of circulating nucleated red cells, suggesting the infection has an impact on stressing erythropoiesis [23].

Regarding erythrocyte parameters, an increase in RDW has been documented in patients suffering from a severe form of COVID-19, compared to subjects with mild disease, also associated with 2.5-fold increased mortality risk. However, we have no evidence of an association between the alteration of this parameter and an increased risk of reinfection with SARS-CoV-2. Furthermore, we cannot define if this is a causal or predisposing factor of the infection, it would be possible to evaluate it only with a cohort study.

In severe cases of COVID-19, a reduction in haemoglobin (Hb) and red blood cell counts has been documented, even without considering the cases of reinfection [24]. In our study, however, an increase in the red blood cell count in cases of reinfection was seen, and they generally have milder, if not completely asymptomatic, clinical pictures compared to the more severe forms of primary infection.

A possible explanation for the increase in the number of red blood cells in cases of reinfection would derive from the observation that SARS-CoV-2 is able to infect red blood cells by damaging their membrane and compromising their functions; in particular, the capacity of blood cells reds to release ATP and to ensure the supply of oxygen to the tissues [25]. The damage induced by the infection would induce the proliferative stimulus of erythroblasts as previously hypothesized. It appears an interesting challenge to understand the relationship between the increase in red blood cells and reinfection.

The appearance of less deformable erythrocytes has been documented in subjects affected by COVID-19 [26]. Cell deformability is a key factor determining splenic clearance and erythrocytes deviating from normal deformability are likely to be removed from the spleen, inducing a proliferative stimulus on erythroblastic colonies. This could help explain the increase in the number of red blood cells we have identified.

The strengths of our work come from the certainty of cases of reinfection with SARS-CoV-2, since they derive from the contact tracing action activated by the Teaching Hospital Policlinico Umberto I in Rome from March 2020.

All the cases of reinfection for which we had the PHD (Personal Health-related Document) were considered.

There were 11 cases of reinfection with SARS-CoV-2 excluded from our work because we were not able to obtain the related medical records as employees of other companies (health service cooperatives; canteen service; fire prevention service etc.) and were therefore subjected to work surveillance visits for occupational medicine at other centers other than the Occupational Medicine Service of the Teaching Hospital Policlinico Umberto I.

The weaknesses of our work include the design of the case-control study and, in particular, the reliability of data relating to habits such as alcohol consumption, tobacco smoking and physical activity as reported by the HCWs. However, the misclassification of exposure to discretionary factors, in particular, the consumption of alcoholic beverages, should not have taken place as the collection of this data is routine during health surveillance visits. Future study must be organized with a cohort design in order to better collect this type of data. 

A further criticality of the study in question could be the lack of data related to the viral genotype between the first and second infection that occurred in cases of reinfection. However, the vast majority of reinfections occurred when the Delta and Omicron variants were spread, and it is likely that this could not have had any impact on our results.

Moreover, as far as socio-demographic factors are concerned, only data concerning occupation was available, while education and income were not. However, a systematic review on the recurrence of SARS-CoV-2, published in 2021, did not consider socio-demographic factors such as income and educational level [27]. 

## 5. Conclusions

The findings in our study allow us to understand that particular attention should be paid to subjects with diabetes mellitus, women and alcoholic drinkers. These categories must be taken into account along with the elderly and patients with comorbidities as possible targets, especially for vaccine-based primary prevention.

These results could also suggest that contact tracing represents a fundamental approach model against SARS-CoV-2 pandemic. Indeed, without it, we would not have been aware of reinfection cases.

Finally, since the evolution of SARS-CoV-2 variants is so rapid, we agree with Deng et al. [28] on the need for continuing to produce reviews and evidence in the near future. 

## Figures and Tables

**Table 1:** Characteristics of the participants.

Variables	N (%) or Mean (SD) [Range]
**Gender**	
Females	250 (62.3)
Males	151 (37.7)
**Age**	43.03 (12.33)
**Profession**	
Physicians	142 (35.3)
Nurses	182 (45.3)
Technicians	10 (2.5)
Administrative	17 (4.2)
Healthcare Assistants	51 (12.7)
**Reinfection**	
Yes	134 (33.4)
No	267 (66.6)
**Smoking**	
No	244 (62.4)
Yes	147 (37.6)
**Audit C**	
0	120 (31.2)
1	105 (27.3)
2	108 (28.1)
3	36 (9.4)
4	11 (2.9)
5	4 (1.0)
6	1 (0.3)
**Physical activity**	
No	173 (44.9)
<4 h per week	146 (37.9)
4 < h < 8 per week	55 (14.3)
>8 h per week	11 (2.9)
**Diabetes**	
No	369 (93.4)
Yes	26 (6.6)
**Hypertension**	
No	337 (86.0)
Yes	55 (14.0)
**Drugs use**	
No	213 (54.6)
Yes	177 (45.4)
**Biological hazard**	
No	14 (3.6)
Yes	378 (96.4)
**Manual handling of loads**	
No	200 (51.0)
Yes	192 (49.0)
**Night shift**	
No	88 (22.6)
Yes	302 (77.4)
**Low-dose ionizing radiations exposure**	
No	336 (85.7)
Yes	56 (14.3)
**BMI**	24.53 (4.68)
**Red blood cells (×10^12^/L)**	4.40 (1.40) [3.77–6.61]
**Haemoglobin (g/dL)**	14.18 (1.38) [8.00–18.03]
**Platelets (×10^9^/L)**	242.71 (98.67) [94–1785]
**White blood cells (×10^9^/L)**	6.04 (2.53) [3.12–13.50]

**Table 2:** Univariate analysis (Reinfection vs. All Controls).

Reinfection vs. All Controls			
Variable	Reinfection (%)	All Controls (%)	*p*
**Gender**			
Females	48 (35.8)	103 (38.6)	0.59
Males	86 (64.2)	164 (61.4)	
**Age**	43.13 (12.51)	42.99 (12.25)	0.91
**Profession**			
Physicians	46 (34.3)	96 (35.8)	0.98
Nurses	62 (46.3)	120 (44.8)	
Technicians	4 (3.0)	6 (2.2)	
Administrative	6 (4.5)	11 (4.1)	
Healthcare Assistants	16 (11.9)	35 (13.1)	
**Smoking**			
No	76 (60.3)	168 (63.4)	0.55
Yes	50 (39.7)	97 (36.6)	
**Audit C**			
0	27 (21.4)	93 (35.9)	0.002
1	29 (23.0)	76 (29.3)	
2	43 (34.1)	65 (25.1)	
3	21 (16.7)	15 (5.8)	
4	4 (3.2)	7 (2.7)	
5	2 (1.6)	2 (0.8)	
6	0 (0.0)	1 (0.4)	
**Physical activity**			
No	61 (48.8)	112 (43.1)	0.48
<4 h per week	42 (33.6)	104 (40.0)	
4 < h < 8 per week	17 (13.6)	38 (14.6)	
>8 h per week	5 (4.0)	6 (2.3)	
**Diabetes**			
No	114 (87.0)	255 (96.6)	<0.001
Yes	17 (13.0)	9 (3.4)	
**Hypertension**			
No	111 (85.4)	226 (86.3)	0.81
Yes	19 (14.6)	36 (13.7)	
**Obesity**			
No	115 (87.8)	238 (90.5)	0.40
Yes	16 (14.6)	25 (9.5)	
**Biological hazard**			
No	6 (4.6)	8 (3.1)	0.43
Yes	124 (95.4)	254 (96.9)	
**Manual handling of loads**			
No	67 (51.5)	133 (50.8)	0.88
Yes	63 (48.5)	129 (49.2)	
**Night shift**			
No	32 (24.8)	56 (21.5)	0.45
Yes	97 (75.2)	205 (78.5)	
**BMI**	24.66 (4.5)	24.45 (4.7)	0.68
**Red blood cells (×10^12^/L)**	4.80 (0.49)	4.17 (1.66)	<0.001
**Haemoglobin (g/dL)**	14.13 (1.18)	14·21 (60.26)	0.56
**Platelets (×10^9^/L)**	245.50 (147.52)	241·30 (60.26)	0.69
**White blood cells (×10^9^/L)**	6.52 (1.74)	5.78 (2.83)	0.007
**Drugs**			
No	72 (55.4)	141 (54.2)	0.82
Yes	58 (44.6)	119 (45.8)	
**Vaccination against COVID-19**			
No	14 (10.4%)	19 (7.1)	0.248
Yes	120 (89.6)	249 (92.9%)	

**Table 3:** Univariate analysis (Reinfection vs. Never Positive).

Reinfection vs. Never Positive			
Variable	Reinfection (%)	Never Positive (%)	*p*
**Gender**			
Females	48 (35.8)	53 (39.3)	0.56
Males	86 (64.2)	82 (60.7)	
**Age**	43.13 (12.51)	43.16 (12.29)	0.98
**Profession**			0.97
Physicians	46 (34.3)	48 (35.6)	
Nurses	62 (46.3)	61 (45.2)	
Technicians	4 (3.0)	3 (2.2)	
Administrative	6 (4.5)	6 (4.4)	
Healthcare Assistants	16 (11.9)	17 (12.6)	
**Smoking**			0.27
No	76 (60.3)	81 (60.0)	
Yes	50 (39.7)	54 (40.0)	
**Audit C**			<0.001
0	27 (21.4)	48 (35.8)	
1	29 (23.0)	44 (32.8)	
2	43 (34.1)	35 (26.1)	
3	21 (16.7)	3 (2.2)	
4	4 (3.2)	3 (2.2)	
5	2 (1.6)	0 (0.0)	
6	0 (0.0)	1 (0.7)	
**Physical activity**			0.63
No	61 (48.8)	65 (48.5)	
<4 h per week	42 (33.6)	46 (34.3)	
4 < h < 8 per week	17 (13.6)	21 (15.7)	
>8 h per week	5 (4.0)	2 (1.5)	
**Diabetes**			<0.001
No	114 (87.0)	134 (99.3)	
Yes	17 (13.0)	1 (0.7)	
**Hypertension**			0.14
No	111 (85.4)	123 (91.1)	
Yes	19 (14.6)	12 (8.9)	
**Obesity**			0.19
No	115 (87.8)	124 (92.5)	
Yes	16 (12.2)	10 (7.5)	
**Biological hazard**			0.95
No	6 (4.6)	6 (4.5)	
Yes	124 (95.4)	128 (95.5)	
**Manual handling of loads**			0.89
No	67 (51.5)	68 (50.7)	
Yes	63 (48.5)	66 (49.3)	
**Night shift**			0.45
No	32 (24.8)	28 (20.9)	
Yes	97 (75.2)	106 (79.1)	
**Ionizing radiations**			0.32
No	109 (83.8)	118 (88.1)	
Si	21 (16.2)	16 (11.9)	
**BMI**	24.66 (4.5)	24·28 (4.76)	0.49
**Red blood cells (×10^12^/L)**	4.80 (0.49)	3·52 (2.13)	<0.001
**Haemoglobin (g/dL)**	14.3 (1.18)	14·23 (1.47)	0.55
**Platelets (×10^9^/L)**	245.50 (147.52)	241·83 (56.21)	0.79
**White blood cells (×10^9^/L)**	6.52 (1.74)	5·06 (3.44)	<0.001
**Drugs**			0.47
No	72 (55.4)	80 (59.7)	
Si	58 (44.6)	54 (40.3)	
**Vaccination against COVID-19**			0.516
No	14 (10.4%)	11 (8.1)	
Yes	120 (89.6)	124 (91.9%)	

**Table 4:** Univariate analysis (Reinfection vs. Single Positivity).

Reinfection vs. Single Positivity			
Variable	Reinfection (%)	Single Positivity (%)	*p*
**Gender**			0.72
Females	48 (35.8)	50 (37.9)	
Males	86 (64.2)	82 (62.1)	
**Age**	43.13 (12.51)	42.80 (12.26)	0.83
**Profession**			0.97
Physicians	46 (343)	48 (36.1)	
Nurses	62 (46.3)	59 (44.4)	
Technicians	4 (3.0)	3 (2.3)	
Administrative	6 (4.5)	5 (3.8)	
Healthcare Assistants	16 (11.9)	18 (13.5)	
**Smoking**			0.27
No	76 (60.3)	87 (66.9)	
Yes	50 (39.7)	43 (33.1)	
**Audit C**			0.09
0	27 (21.4)	45 (36.0)	
1	29 (23.0)	32 (25.6)	
2	43 (34.1)	30 (24.0)	
3	21 (16.7)	12 (9.6)	
4	4 (3.2)	4 (3.2)	
5	2 (1.6)	2 (1.6)	
6	0 (0.0)	0 (0.0)	
**Physical activity**			0.21
No	61 (48.8)	47 (37.3)	
<4 h per week	42 (33.6)	58 (46.0)	
4 < h < 8 per week	17 (13.6)	17 (13.5)	
>8 h per week	5 (4.0)	4 (3.2)	
**Diabetes**			0.064
No	114 (87.0)	121 (93.8)	
Yes	17 (13.0)	8 (6.2)	
**Hypertension**			0.35
No	111 (85.4)	103 (81.1)	
Yes	19 (14.6)	24 (18.9)	
**Obesity**			0.88
No	115 (87.8)	114 (88.4)	
Yes	16 (12.2)	15 (11.6)	
**Biological hazard**			0.15
No	6 (4.6)	2 (1.6)	
Yes	124 (95.4)	126 (98.4)	
**Manual handling of loads**			0.90
No	67 (51.5)	65 (50.8)	
Yes	63 (48.5)	63 (49.2)	
**Night shift**			0.60
No	32 (24.8)	28 (22.0)	
Yes	97 (75.2)	99 (78.0)	
**Ionizing radiations**			0.77
No	109 (83.8)	109 (85.2)	
Si	21 (16.2)	19 (14.8)	
**BMI**	24.66 (4.5)	24.64 (4.80)	0.97
**Red blood cells (×10^12^/L)**	4.80 (0.49)	4.81 (0.46)	0.87
**Haemoglobin (g/dL)**	14.13 (1.18)	14.20 (1.48)	0.66
**Platelets (×10^9^/L)**	245.50 (147.52)	240.74 (64.53)	0.74
**White blood cells (×10^9^/L)**	6·52 (1.74)	6.52 (1.77)	0.99
**Drugs**			0.26
No	72 (55.4)	61 (48.4)	
Si	58 (44.6)	65 (51.6)	
**Vaccination against COVID-19**			0.188
No	14 (10.4%)	8 (6)	
Yes	120 (89.6)	125 (914%)	

**Table 5:** Multivariate analysis (Reinfection vs. All Controls).

Reinfection vs. All Controls		
	Reinfection	All Controls
Variable	Full Model OR [IC 95%]	Stepwise Model OR [IC 95%]
**Gender**		
Males (ref.)	1	1
Females	2.05 (1.01–4.20)	2.42 (1.38–4.25)
**Age**	1.01 (0.99–1.04)	
**BMI**	0.98 (0.92–1.05)	
**Profession**		
Physicians	0	
Nurses	0	
Technicians	0	
Administrative	0	
Healthcare Assistants	0	
Other healthcare workers (ref)	1	
**Drugs**		
Yes	0.75 (0.42–1.34)	
No (ref)	1	
**Smoking**		
Yes		
No (ref)	1.25 (0.74–2.11)	
**Audit C**	1.53 (1.20–1.95)	1.49 (1.19–1.87)
**Diabetes**		3.45 (1.41–8.46)
Yes	5.82 (1.99–17.06)	
No (ref)	1	
**Hypertension**		
Yes	0.58 (0.21–1.64)	
No (ref)	1	
**Weekly physical activity**	0.99 (0.91–1.37)	
**Red blood cells (×10^12^/L)**	1.99 (1.35–2.94)	1.69 (1.21–2.25)
**Haemoglobin g/dL**	0.85 (0.67–1.08)	
**White blood cells (×10^9^/L)**	0.91 (0.79–1.05)	

**Table 6:** Multivariate analysis: Reinfection vs. Never Positive.

Reinfection vs. Never Positive		
	Reinfection	Never Positive
Variable	Full Model OR [IC 95%]	Stepwise Model OR [IC 95%]
**Gender**		
Males (ref.)	1	1
Females	2.96 (1.22–7.22)	3.42 (1.66–7.03)
**Age**	1.00 (0.98–1.04)	
**BMI**	1.00 (0.92–1.09)	
**Profession**		
Physicians	0	
Nurses	0	
Technicians	0	
Administrative	0	
Healthcare Assistants	0	
Other healthcare workers (ref)	1	
**Drugs**		
Yes	0.76 (0.38–1.54)	
No (ref)	1	
**Smoking**		
Yes	1.16 (0.61–2.22)	
No (ref)	1	
**Audit C**	1.58 (1.14–2.18)	1.51 (1.13–2.01)
**Diabetes**		
Yes	16.10 (1.88–137.58)	15.42 (1.92–123.6)
No (ref)	1	
**Hypertension**		
Yes	1.58 (0.39–6.46)	
No (ref)	1	
**Weekly physical activity**	1.07 (0.72–1.57)	
**Red blood cells (×10^12^/L)**	2.66 (1.69–4.20)	2.47 (1.63–3.74)
**Haemoglobin g/dL**	0.88 (0.65–1.19)	
**White blood cells (×10^9^/L)**	0.84 (0.70–1.02)	

**Table 7:** Multivariate analysis: Reinfection vs. Single Positivity.

Reinfection vs. Single Positivity		
	Reinfection	Single Positivity
Variable	Full Model OR [IC 95%]	Stepwise Model OR [IC 95%]
**Gender**		
Males (ref.)	1	
Females	1.48 (0.63–3.46)	
**Age**	1.01 (0.98–1.04)	
**BMI**	0.98 (0.91–1.06)	
**Profession**		
Physicians	0	
Nurses	0	
Technicians	0	
Administrative	0	
Healthcare Assistants	0	
Other healthcare workers (ref)	1	
**Drugs**		
Yes	0.70 (0.35–1.38)	
No (ref)	1	
**Smoking**		
Yes	1.31 (0.72–2.41)	
No (ref)	1	
**Audit C**	1.54 (1.16–2.04)	1.40 (1.09–1.79)
**Diabetes**		
Yes	4.51 (1.40–14.61)	4.39 (1.41–13.67)
No (ref)	1	1
**Hypertension**		
Yes	0.33 (0.10–1.08)	0.28 (0.11–0.74)
No (ref)	1	1
**Weekly physical activity**	0.83 (0.56–1.23)	
**Red blood cells (×10^12^/L)**	1.09 (0.49–2.43)	
**Haemoglobin g/dL**	0.91 (0.67–1.25)	
**White blood cells (×10^9^/L)**	0.96 (0.80–1.14)	

## Data Availability

Data are available upon request to the authors.
